# AI Agents and the Future of Clinical Judgment in Medical Education: Opportunities, Challenges, and the Need for Human‐Centered Integration

**DOI:** 10.1002/hsr2.73042

**Published:** 2026-08-12

**Authors:** Soleiman Ahmady, Noushin Kohan, Alireza Monajemi

**Affiliations:** ^1^ Department of Medical Education, School of Medical Education and Learning Technologies Shahid Beheshti University of Medical Sciences Tehran Iran; ^2^ Research affilited faculty, Department of LIME Karolinska Institutet Stochholm Sweden; ^3^ Department of Medical Education and E‐learning in Medical Education Smart University of Medical Sciences Tehran Iran; ^4^ Department of History and Philosophy of Science Institute for Humanities and Cultural Studies Tehran Iran

**Keywords:** AI agents, AI literacy, artificial intelligence, clinical judgment, clinical reasoning, generative artificial intelligence, health professions education, large language models, medical education, responsible AI

## Abstract

**Background:**

Artificial intelligence (AI) is actively transforming health professions education by introducing innovative methodologies for learning, simulation, and clinical decision support. The recent emergence of autonomous AI agents—equipped with advanced capabilities like memory, planning, and tool integration—creates unprecedented opportunities for medical training. However, this growing technological autonomy simultaneously introduces critical challenges regarding clinical judgment, professional accountability, and educational equity.

**Methods:**

This perspective employs a rigorous critical analysis grounded in health professions education, medical philosophy, and AI ethics literature. It systematically evaluates the pedagogical potentials alongside the epistemological risks associated with deploying autonomous AI agents within clinical training environments.

**Discussion:**

While AI agents can effectively drive adaptive learning, scalable simulations, and individualized feedback, unmonitored or inappropriate integration risks undermining core clinical reasoning. Over‐reliance on algorithmic suggestions can diminish a learner's independent analytical capabilities and propagate inherent data set biases. Conversely, dismissing these technologies risks depriving learners of vital digital competencies. Consequently, the ultimate educational utility of AI agents is fundamentally dictated by deliberate instructional design and robust governance.

**Conclusion:**

Rather than serving as replacements for human expertise, AI agents must be harnessed through a human‐centered framework that safeguards clinical reasoning, ethical reflection, and professional accountability. By deploying targeted strategies—such as foundational AI literacy, localized data governance, controlled simulation failures, and modernized evaluations like AI‐assisted OSCEs—educational institutions can ensure these technologies reinforce human‐driven medical practice.

## 1 AI Agents and the Emerging Paradigm in Medical Education

AI agents constitute a transformative frontier in health professions education, driven by sophisticated architectures that integrate memory, planning, and tool interaction. Operating as an evolutionary extension of foundational language models, these advanced functionalities empower immersive modalities, including adaptive learning pathways, virtual patient simulations, and tailored feedback mechanisms [1].

Nevertheless, incorporating autonomous AI agents into medical curricula mandates a rigorous evaluation of their downstream effects on clinical judgment. Rather than adopting an alarmist lens or uncritical techno‐optimism, scholarship must weigh genuine pedagogical affordances against their epistemological risks [2].

Clinical expertise encompasses far more than knowledge retrieval; it demands nuanced contextual interpretation, ethical reasoning, effective communication, and the management of clinical uncertainty (phronēsis)—an essential dimension of professional wisdom that cannot be fully automated. Although AI agents can amplify learning acquisition and reflection, uncritical dependence on algorithmic recommendations directly compromises independent reasoning and diagnostic decision‐making. Furthermore, structural biases embedded within training data sets fundamentally skew algorithmic outputs unless subjected to systematic critical appraisal [3].

## 2 AI Agents Versus Large Language Models: Educational Implications

Large language models (LLMs) and AI agents represent successive evolutionary milestones in artificial intelligence, with distinct implications for medical education. Conventional LLMs function primarily as reactive text generators based on pattern recognition. While they offer valuable knowledge, explanation, and learning support, their inherent inaccuracies and contextual limitations simultaneously provide opportunities for learners to practice critical appraisal and verification [4].

AI agents, conversely, build upon LLM foundations by incorporating advanced capabilities such as memory, planning, goal‐directed actions, and seamless integration with external tools (e.g., clinical databases, guidelines, and simulation environments). This transition shifts technology from passive text generation to adaptive, autonomous educational engagement [5].

From a medical education perspective, this distinction is critical because clinical competence requires active interpretation, hypothesis generation, and uncertainty management rather than mere information access. While AI agents facilitate advanced adaptive learning, virtual patients, and individualized feedback, their heightened autonomy introduces a major educational hazard: if learners delegate core cognitive tasks to the system, opportunities for independent clinical reasoning and practical wisdom (phronēsis) are severely diminished [6].

The conceptual divergences between LLMs and AI agents, alongside their respective educational affordances and risks, are systematically detailed in Table 1 [1, 5, 6]. As outlined in the table, while AI agents foster personalized and interactive learning, careful instructional design is mandatory to ensure learners remain actively engaged in deep cognitive processing. Ultimately, AI agents must be integrated strictly as cognitive scaffolds that support human expertise rather than systems that replace the cognitive processes required for clinical excellence.

**Table 1:** Distinction between Large Language Models and AI Agents and Their Educational Implications.

Feature	Large Language Models (LLMs)	AI agents
Core function	Generate and transform language‐based responses based on learned patterns from large data sets.	Extend LLM foundations through memory, planning, goal‐directed actions, and external tool interaction.
Interaction with learners	Primarily provide responses, explanations, and learning support based on user prompts.	Enable adaptive, sustained, and interactive engagement through autonomous educational tasks.
Tool integration	Usually limited or restricted unless explicitly connected to external applications.	Actively interact with clinical databases, clinical guidelines, and simulation environments.
Educational applications	Knowledge support, explanation, question generation, and basic learning assistance.	Adaptive tutoring, virtual patient simulations, simulation‐based learning, and individualized feedback.
Potential educational value	Encourages exploration, digital literacy, and critical evaluation of AI‐generated text.	Supports deliberate practice, immersive reflection, and highly personalized learning trajectories.
Potential concerns & cognitive risks	Inaccurate outputs, hallucinations, and inappropriate reliance on unverified text.	Reduction in independent reasoning and diagnostic engagement if core cognitive tasks are fully delegated to autonomous systems.

## 3 Ethical and Epistemological Considerations

1. AI Agents and the Development of Clinical Judgment
  Clinical judgment requires contextual interpretation and the management of uncertainty, deeply rooted in practical wisdom (phronēsis)—an essential human dimension that cannot be automated. While AI agents offer diverse practice scenarios, uncritical reliance on their outputs risks stunting independent diagnostic reasoning. Educational frameworks must therefore position AI as a catalyst for active critical reflection, compelling learners to interrogate underlying assumptions rather than passively accept algorithmic suggestion [7].

2. Bias, Equity, and Algorithmic Awareness
  AI systems risk reproducing historical inequalities and systemic biases embedded within training data sets. While localized data governance is often proposed as a remedy, it presents a structural paradox, as local data sets themselves frequently suffer from incomplete records and hidden skews. Consequently, developing robust AI literacy—enabling learners to critically dissect data limitations and algorithmic assumptions—is vital for responsible integration [8].

3. Accountability, Human Responsibility, and Legal Liability
  The growing autonomy of AI agents creates severe ambiguities regarding transparency, institutional oversight, and legal liability in cases of medical malpractice. Professional accountability and ethical responsibility toward patients must unequivocally remain with human practitioners and educators. Institutions must establish clear regulatory frameworks and explicit protocols that treat AI outputs strictly as interpretive suggestions rather than definitive conclusions [9].

## 4 Practical Recommendations for Responsible Integration of AI Agents in Medical Education

**Figure 1:**
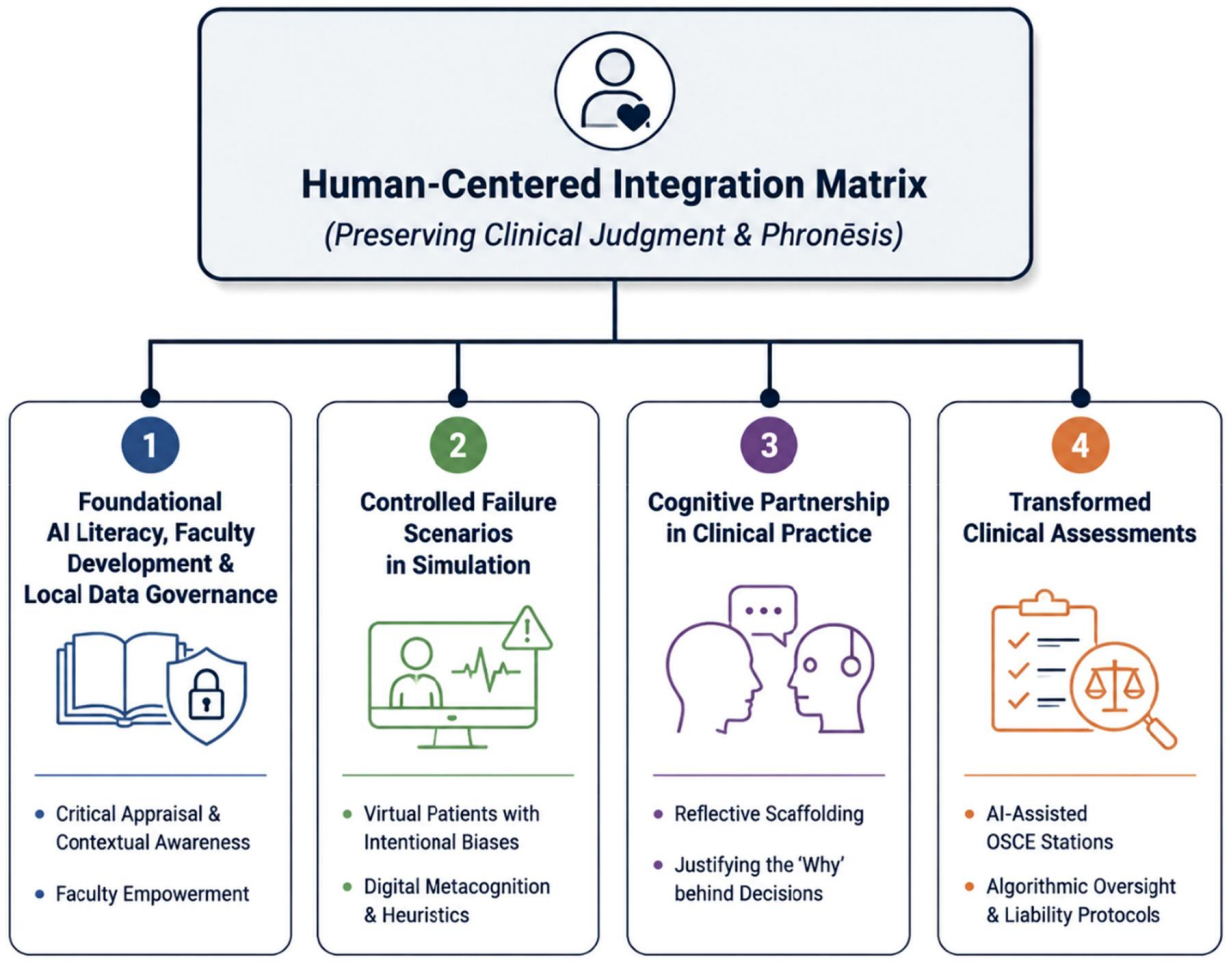
. Human‐Centered Integration Matrix illustrating the four interconnected dimensions—foundational literacy and governance, failure‐based simulation, cognitive partnership, and modernized assessment—designed to protect and reinforce independent clinical judgment (phronēsis) in the AI era.

To operationalize the integration of AI agents without compromising clinical judgment, medical institutions and educators should adopt a human‐centered matrix comprising four core dimensions, as illustrated in Figure 1.

First, institutions must prioritize Foundational AI Literacy, Faculty Development, and Local Data Governance. Recognizing that clinical educators themselves frequently lack digital proficiency, institutions must mandate faculty empowerment programs alongside training students in algorithmic logic, data privacy, and critical appraisal. Furthermore, trainees must be guided to evaluate AI outputs against regional epidemiological realities while remaining critically aware of the structural limitations and hidden flaws inherent in local healthcare data sets [10].

Second, simulation‐based training should incorporate AI‐supported virtual patients and cases that expose learners to diagnostic uncertainty and variable clinical scenarios. Such approaches can promote critical appraisal of AI‐generated information, strengthen digital metacognition, and help learners recognize the limitations of algorithmic recommendations rather than relying on them uncritically [11].

Third, AI agents should be conceptualized as cognitive partners in clinical learning, serving as reflective scaffolds that encourage learners to articulate the rationale behind clinical decisions, evaluate alternative interpretations, and critically engage with AI‐generated recommendations rather than passively accepting them [12].

Finally, clinical assessments should evolve to incorporate AI‐supported learning environments that evaluate not only clinical knowledge and decision‐making skills but also learners' ability to critically interpret, validate, and appropriately integrate AI‐generated information within patient‐centered care. Such assessments should be accompanied by clear institutional frameworks to ensure accountability, safety, and responsible use of AI in clinical practice [12].

## 5 Conclusion

Ultimately, AI agents represent a significant opportunity to advance medical education through adaptive learning, personalized feedback, and immersive simulation. However, their integration requires careful educational design to ensure that technological support enhances rather than displaces the development of independent clinical judgment, reflective practice, and practical wisdom (phronēsis). As argued in this perspective, the central challenge is not whether AI should be incorporated into medical education, but how it can be integrated while preserving the cognitive, ethical, and human foundations of clinical decision‐making.

Addressing challenges related to algorithmic bias, data limitations, accountability, and appropriate human oversight requires a shift from passive adoption toward intentional institutional transformation. Through structured approaches—including AI literacy education, faculty development, responsible data governance, simulation‐based critical engagement, and AI‐informed assessment strategies—medical education can position AI agents as cognitive partners that augment clinical reasoning and support the development of competent, reflective, and ethically grounded healthcare professionals.

## Author Contributions

**Soleiman Ahmady:** conceptualization, data curation, methodology, writing – review and editing, project administration. **Noushin Kohan:** writing – review and editing, supervision, conceptualization. **Alireza Monajemi:** validation, supervision.

## Funding

The authors have nothing to report.

## Ethics Statement

This article is a Perspective based on critical analysis of published literature and theoretical frameworks and does not involve any human subjects or data collection. Therefore, formal ethics approval and consent to participate are Not Applicable.

## Conflicts of Interest

The authors declare no conflicts of interest.

## Transparency Statement

The corresponding author (Dr Noushin Kohan) affirms that this manuscript is an honest, accurate, and transparent account of the study being reported.

## Data Availability

Data sharing is not applicable to this article as no data sets were generated or analyzed during the current study.
